# Preoperative predictive factors for opaque bubble layer formation and area during small-incision lenticule extraction: predictive models based on machine learning

**DOI:** 10.1186/s40662-026-00509-w

**Published:** 2026-09-21

**Authors:** Chuzhi Peng, Xi Chen, Ying Yang, Huanhuan Ren, Yuke Huang, Jin Qiu, Yan Li, Keming Yu, Jing Zhuang

**Affiliations:** Zhongshan Ophthalmic Center, WHO Collaborating Center for Eye Care and Vision, State Key Laboratory of Ophthalmology, Guangdong Provincial Key Laboratory of Ophthalmology and Visual Science, Guangdong Provincial Clinical Research Center for Ocular Diseases, Sun Yat-sen University, No.7 Jinsui Road, Tianhe District, Guangzhou, 510060 China

**Keywords:** Machine learning, Opaque bubble layer, Small-incision lenticule extraction, Predictive factors

## Abstract

**Background:**

We aimed to develop exploratory machine learning (ML)-based predictive models for the occurrence and area of an opaque bubble layer (OBL) during small-incision lenticule extraction (SMILE) and identify associated preoperative and surgical-planning factors.

**Methods:**

This retrospective study included 216 eyes (72 with an OBL, 144 controls) that underwent SMILE at the Zhongshan Ophthalmic Center between August 2024 and August 2025, which were matched 1:2 by age and sex. Comprehensive preoperative ocular examinations were performed using a Pentacam HR camera system and standard equipment. The OBL area was quantified from intraoperative videos using ImageJ software. Fifty-four preoperative and surgical features were used to develop the ML models: 15 classification algorithms for OBL occurrence, and 19 regression algorithms for the relative OBL area. Shapley additive explanations analysis was applied for model interpretability.

**Results:**

The Extra Trees model achieved optimal performance for predicting OBL occurrence (area under the receiver operating characteristic curve = 0.885, accuracy = 0.820). The top five key factors included femtosecond laser energy, intraocular pressure, residual stromal thickness, 10-mm corneal volume, and total astigmatism, all of which showed positive correlations. For OBL area prediction, the random forest regression model performed best in the test sets (mean absolute error = 2.89%, root mean square error = 3.37%). Corneal optical density (within the central 2-mm zone of the anterior 120-μm corneal layer) and age were negatively associated with OBL area, whereas keratoconus index showed the strongest positive association.

**Conclusion:**

The ML models showed exploratory predictive ability for OBL occurrence and area during SMILE using preoperative and surgical-planning parameters. External validation and recalibration in consecutive cohorts with a natural OBL prevalence are required before clinical use.

**Supplementary Information:**

The online version contains supplementary material available at https://doi.org/10.1186/s40662-026-00509-w.

## Background

Uncorrected refractive error remains a leading cause of global vision impairment [1], imposing a growing societal and public-health burden. With advancements in modern ophthalmology, corneal refractive surgery has emerged as a significant approach for correcting refractive errors, such as myopia and astigmatism [2]. Among these procedures, small-incision lenticule extraction (SMILE) has gained widespread clinical adoption due to its advantages of being minimally invasive and flap free, as well as preserving corneal biomechanical stability [2–4]. However, intraoperative complications occurring during SMILE can compromise surgical outcomes and safety. Notably, the formation of an opaque bubble layer (OBL) may lead to difficult lenticule dissection and tears [5, 6].

OBL formation results from the photodisruption effect of the femtosecond laser, which generates intrastromal gas bubbles that cannot be immediately evacuated [7–9]. Their accumulation creates an opaque zone, thereby obscuring the visualization of deeper corneal structures during surgery and increasing the difficulty of lenticule dissection and the risk of lenticule tears. Previous studies have preliminarily investigated the associations between OBL occurrence and parameters such as corneal and cap thicknesses [6, 10–12]. However, existing studies have mainly focused on a limited number of clinical or surgical variables using conventional statistical approaches, which may not fully capture the nonlinear or interactive relationships among corneal morphology, optical quality, intraocular pressure (IOP), and surgical-planning parameters. In addition, most previous studies have focused on OBL occurrence, whereas quantitative predictions of OBL extent and the role of broader preoperative parameters, such as corneal densitometry, remain poorly explored. Predicting OBL occurrence and area has related but distinct clinical implications. OBL occurrence identifies eyes in which an intraoperative OBL may develop and thus supports preoperative risk awareness. In contrast, the relative OBL area reflects the extent of bubble-layer involvement, which may be more closely related to surgical visibility, lenticule dissection difficulty, and the risk of OBL-related intraoperative complications. Therefore, quantitative prediction of the OBL area may provide additional information beyond binary OBL occurrence and help refine future risk-stratification strategies.

**Fig. 1:**
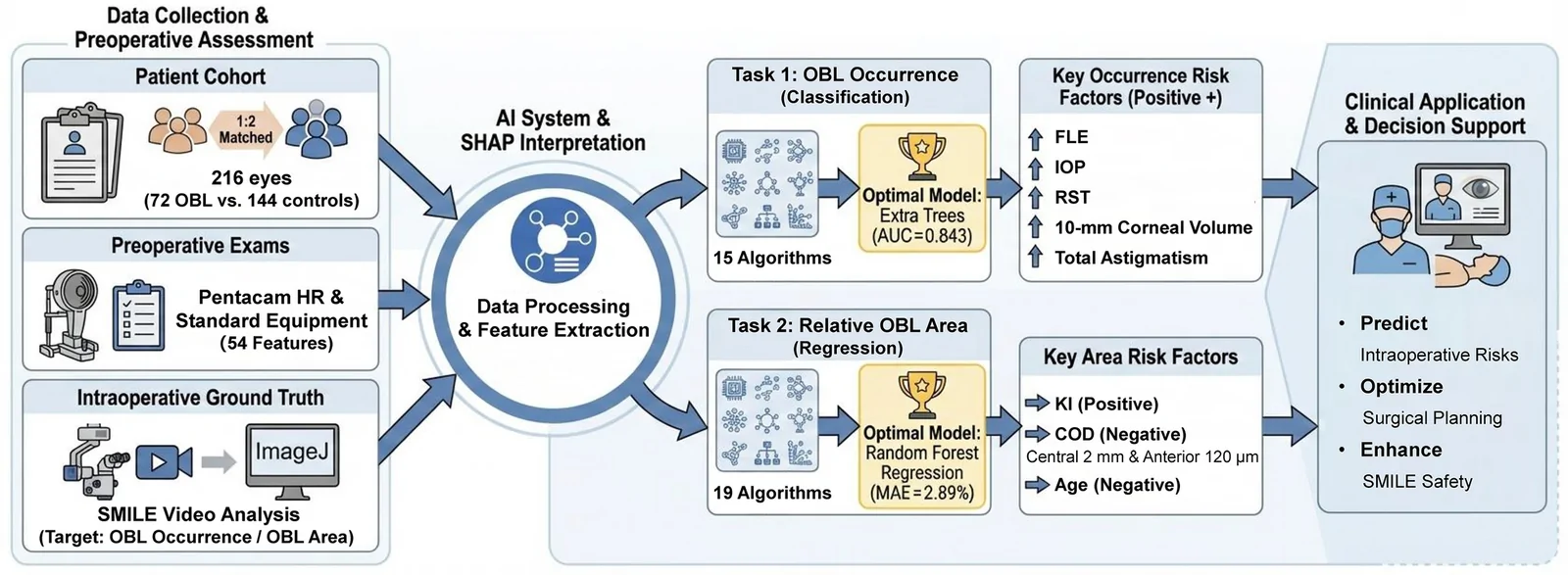
Schematic illustration of the workflow for predicting the risk and relative area of intraoperative OBL. AUC, area under the receiver operating characteristic curve; COD, corneal optical density; FLE, femtosecond laser energy; IOP, intraocular pressure; KI, keratoconus index; MAE, mean absolute error; OBL, opaque bubble layer; RST, residual stromal thickness; SHAP, Shapley additive explanations; SMILE, small-incision lenticule extraction

To address these gaps, this study aimed to develop an exploratory two-stage machine-learning (ML) framework to predict OBL occurrence and the relative OBL area in myopic and/or astigmatic candidates undergoing SMILE while identifying associated preoperative and surgical-planning factors (Fig. 1). Based on our clinical observations and the methodologies adopted in previous studies [6, 11, 13, 14], we specifically focused on an OBL generated during the posterior lenticule cut, as an OBL occurring at this stage is of greater clinical relevance. The application is intended to provide preliminary evidence for preoperative risk stratification.

## Methods

### Study design and population

This retrospective study was conducted at the Zhongshan Ophthalmic Center, Sun Yat-sen University (Guangzhou, China), from August 2024 to August 2025. All eyes underwent SMILE for the correction of myopia and/or astigmatism, and all surgical procedures were performed by a single, experienced ophthalmic surgeon to eliminate inter-surgeon variability.

**Fig. 2:**
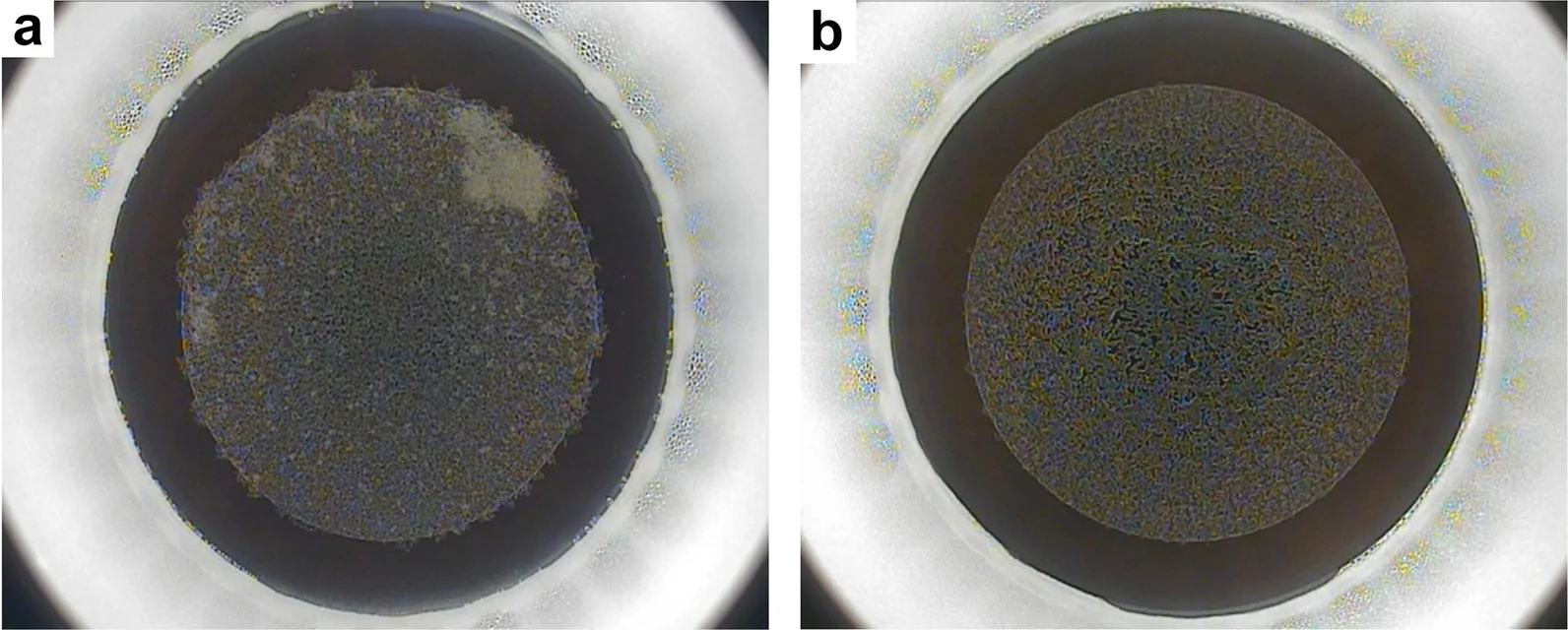
Representative images illustrating group classification based on intraoperative posterior lenticule scanning during SMILE. **a** OBL group; **b** Control group. OBL, opaque bubble layer; SMILE, small-incision lenticule extraction

An opaque bubble layer (OBL) was defined as a visible, opaque intrastromal gas-bubble layer during posterior lenticule scanning. Eyes were classified into the OBL group (case group, 72 eyes; Fig. 2a) when a clearly visible OBL was present and involved the posterior lenticule interface. Control eyes were defined as eyes with no visible OBL or only minimal scattered bubbles that did not form a continuous, opaque layer (Fig. 2b). The control group was enrolled at a 1:2 ratio and matched with the OBL group for age and sex to minimize confounding by these factors. Only one eye of each patient was included in the analysis. Therefore, the final dataset consisted of 216 eyes derived from 216 patients, and inter-eye correlation was not applicable.

The inclusion criteria were: (1) age 18–45 years; (2) spherical equivalent (SE) from −1.00 D to −10.00 D and cylinder ≤ 6.00 D; (3) no prior corneal refractive or ocular surgery; (4) no active ocular disease (e.g., keratoconus, dry-eye syndrome with severe ocular surface damage, and uveitis); and (5) complete preoperative examination data and high-quality intraoperative surgical video recordings. The exclusion criteria were: (1) poor-quality surgical videos from which the OBL area could not be quantified; (2) intraoperative complications other than an OBL; and (3) incomplete preoperative clinical data.

All study procedures were conducted in accordance with the World Medical Association’s Declaration of Helsinki tenets. Ethical approval was obtained from the Ethics Committee of the Zhongshan Ophthalmic Center (Approval No. 2023KYPJ332). The requirement for informed consent was waived by the Ethics Committee owing to the retrospective nature of the study.

### Preoperative ocular examinations

All participants underwent a comprehensive preoperative ophthalmic examination, and all measurements were obtained by trained technicians using standardized equipment to ensure consistency. All corneal morphological and optical parameters were measured using Pentacam HR Scheimpflug tomography (Oculus Optikerate; Carl Zeiss, Oberkochen Germany).

The examined parameters were categorized as follows. (1) Demographic and refractive parameters: age, gender, SE, total astigmatism, and axial length (AL); (2) IOP: measured with a noncontact tonometer; (3) corneal parameters: corneal thickness, corneal volume (CV) in various diameter ranges (3, 5, 7, and 10 mm), corneal curvature [anterior/posterior flat keratometry (K1) and steep keratometry (K2)], corneal irregularity [e.g., index of surface variance (ISV), keratoconus index (KI), and corneal optical density (COD), measured in different corneal regions and layers], and corneal asphericity (e.g., anterior surface eccentricity); and (4) surgical-planning parameters: corneal cap diameter and thickness, femtosecond laser energy, and residual stromal thickness (RST).

### OBL assessment and quantitative measurement

OBL was defined as the formation of an opaque gas-bubble layer in the corneal stroma during femtosecond laser scanning for lenticule creation. Quantitative measurement of the OBL area was performed using ImageJ software (National Institutes of Health, Bethesda, MD, USA). A representative single-frame image was extracted from the intraoperative surgical video at the completion of posterior lenticule surface scanning, corresponding to the time point at which the OBL was most prominent and the posterior lenticule surface boundary clearly identifiable. After conversion to 8-bit grayscale, a single uniform threshold value was selected during a pilot assessment of representative images by comparing the threshold OBL regions with the visible OBL boundaries on the original intraoperative frames. This threshold was then consistently applied to all images without image-specific adjustment. The relative OBL area was calculated as the primary quantitative index. OBL area measurements were performed by observers who were masked to the patients’ clinical data and model-predictor variables.

### Feature selection and machine-learning model development

#### Data preprocessing

A total of 54 candidate preoperative and surgical features were initially considered for model development, covering demographic, refractive, corneal morphological, corneal optical, IOP, and surgical parameters. Age and sex were not used as predictors for the OBL occurrence classification task because they defined the matching procedure. The conditional regression task was restricted to OBL-positive eyes, and age remained an eligible predictor in that task. Using PyCaret (version 3.3.2), an automated ML framework with built-in classification and regression model libraries, with train_size = 0.80, the classification dataset of 216 eyes was divided into 172 eyes for training and cross-validation and 44 eyes for internal hold-out testing; the split was stratified by OBL status. The conditional regression dataset of 72 OBL-positive eyes was divided into 57 eyes for training and cross-validation and 15 eyes for internal hold-out testing. All preprocessing steps were fitted within the corresponding training set and then applied to its internal test set. Continuous variables were standardized using z-score normalization based on the mean and standard deviation (SD) of the training set. Multicollinearity filtering was also fitted within the training set. Hyperparameter tuning and model selection were exclusively performed within the training set using 10-fold cross-validation; no separate validation cohort was created, and the internal hold-out test set used only for the final evaluation. As complete preoperative examination data were required for inclusion, no missing values remained in the final analytical dataset, and no imputation performed. No additional class weighting, oversampling, or undersampling was applied.

#### Model construction

To reduce feature redundancy and multicollinearity among the corneal structural and tomographic parameters, a correlation-based multicollinearity filtering procedure was applied before model training. Pairwise correlations among candidate predictors were evaluated, and when the absolute correlation coefficient exceeded 0.90, redundant variables were removed according to the prespecified feature-filtering procedure. This step was implemented to reduce instability in model training and improve the interpretability of feature-importance analyses.

Model development was performed using PyCaret (version 3.3.2), an automated ML framework with built-in classification and regression model libraries. The candidate models included common linear, kernel-based, nearest-neighbor, naive Bayes, decision-tree, ensemble, and gradient-boosting algorithms. Hyperparameters were tuned with the tune_model function of PyCaret using its model-specific predefined search distributions and default scikit-learn search backend. Under the PyCaret defaults used here, the procedure performs a randomized search over 10 candidate configurations (n_iter = 10), unless the predefined space contains ≤10 possible combinations, in which case all combinations are evaluated automatically. Thus, we did not define or perform a manually specified exhaustive grid search. Fifteen classification algorithms were evaluated for the binary outcome of OBL occurrence, and 19 regression algorithms evaluated for the continuous outcome of the relative OBL area [15].

Each candidate algorithm and its tuned configurations were evaluated through 10-fold cross-validation within the task-specific training set. The term “training-set 10-fold cross-validation” refers to performance on the held-out fold in each of the 10 rotations aggregated across folds; it does not denote a separately reserved validation dataset. After tuning and selection, the fitted model pipeline was evaluated once on the untouched internal hold-out test set. A two-stage modeling framework was adopted. In the first stage, all included eyes were used to develop classification models for predicting OBL occurrence. In the second stage, regression models were developed only among eyes with an OBL to predict the relative OBL area conditional on OBL occurrence. Therefore, control eyes with no or a barely detectable OBL were not included in the regression task to avoid direct modeling of a zero-inflated outcome distribution. Baseline comparisons between the OBL and control groups were performed for descriptive purposes only and not used for feature selection or model-input determination.

#### Model evaluation metrics

For the classification models of OBL occurrence, the primary evaluation metric was the area under the receiver operating characteristic curve (AUC), and accuracy (ACC) reported as a secondary metric. For regression models of the relative OBL area, the mean absolute error (MAE) and root mean square error (RMSE) were used to summarize the prediction-error magnitude. Point estimates and 95% confidence intervals (CIs) were reported for performance on the internal hold-out test sets. Given the modest internal hold-out samples, these intervals were interpreted as conditional, descriptive estimates rather than as uncertainty for the full model-development process. The bootstrapping procedure is described in the “Statistical analysis” section.

#### Model interpretability

Shapley additive explanations (SHAP; version 0.41.0) was applied to the optimal classification model for further interpretability analysis. A SHAP summary plot was generated to quantify the global feature importance and direction of the association (positive/negative) between each feature and the risk of OBL occurrence or a larger OBL area.

### Statistical analysis

Statistical analyses were performed using R (version 4.4.2; The R Foundation, Vienna, Austria) and Python (version 3.13.0; Python Software Foundation, Wilmington, DE, USA). Data were checked for normality and homogeneity of variance using the Shapiro–Wilk and Levene’s tests. Normally distributed data are presented as mean ± SD; non-normally distributed data are presented as median (interquartile range) and were compared using the Mann–Whitney U test. Categorical variables are presented as numbers (percentages) and were compared using the chi-square test. A two-sided *P* < 0.05 was considered statistically significant.

For performance on the internal hold-out test sets, 95% CIs were estimated using 2000 nonparametric bootstrap resamples of the fixed test-set predictions. In each iteration, the paired observed outcomes and model predictions were resampled with replacement at the eye level using a bootstrap sample equal in size to that of the corresponding internal test set. For classification, resampling was performed within the observed outcome classes such that both OBL-positive and control eyes were represented in every iteration, and the AUC and ACC recalculated. For the conditional regression, the 15 observed-predicted pairs were resampled with replacement, and the MAE and RMSE recalculated. The 2.5th and 97.5th percentiles of the bootstrap distributions were used as the 95% confidence limits. Bootstrapping was applied only to predictions from the fixed internal hold-out test sets; preprocessing, model fitting, hyperparameter tuning, and model selection were not repeated in each bootstrap iteration. Therefore, the CIs quantify test-set sampling variability conditional on the selected models and the specific train-test splits and do not incorporate uncertainty from the full model-development and selection process.

## Results

### Baseline clinical and ocular characteristics of the study population

A total of 216 eyes were included in the study (72 eyes in the OBL group and 144 in the control group). No statistically significant differences were observed in age or sex between the two groups (all *P* > 0.05), confirming the effectiveness of matching. Compared with the control group, the OBL group had a significantly higher IOP (*P* < 0.001), thicker RST (*P* < 0.001), higher femtosecond laser energy (*P* < 0.001), lower magnitude of total astigmatism (*P* < 0.001), smaller corneal astigmatism (*P* = 0.022), smaller anterior surface numerical eccentricity (eccentricity F, *P* = 0.026), larger posterior K1 (*P* = 0.005) and K2 (*P* = 0.025), larger CV in various diameter ranges (3, 5, 7, and 10 mm; all *P* < 0.001), and smaller ISV (*P* = 0.011). The statistically significant results are shown in Table 1, and detailed baseline data presented in Additional file 1. We randomly divided 216 eyes, allocating 80% to the training and cross-validation set and the remaining 20% to an internal hold-out test set for model evaluation.

**Table 1:** Ocular characteristics data with statistical significance between the OBL group and its controls

Variables	Control	OBL	*P* value
IOP (mmHg)	14.06 ± 2.23	15.99 ± 2.95	< 0.001
RST (μm)	321.58 ± 25.68	340.67 ± 34.94	< 0.001
FLE index	27.14 ± 0.35	27.60 ± 0.49	< 0.001
Total astigmatism (D)	−1.01 ± 0.77	−0.68 ± 0.77	< 0.001
Corneal astigmatism F (D)	−1.36 ± 0.74	−1.13 ± 0.64	0.022
Eccentricity F (mm)	0.55 ± 0.11	0.52 ± 0.11	0.026
ISV	18.01 ± 5.02	16.15 ± 4.55	0.011
K1 B (D)	−6.04 ± 0.19	−6.14 ± 0.23	0.005
K2 B (D)	−6.41 ± 0.23	−6.49 ± 0.23	0.025
Corneal volume (mm^3^)			
Central 3 mm	3.96 ± 0.17	4.09 ± 0.22	< 0.001
Central 5 mm	11.58 ± 0.49	11.98 ± 0.62	< 0.001
Central 7 mm	24.90 ± 1.06	25.78 ± 1.28	< 0.001
Central 10 mm	61.00 ± 2.73	63.25 ± 3.03	< 0.001
Relative OBL area (%)	0.06 ± 0.05	7.66 ± 3.59	< 0.001

*B* = posterior; *F* = anterior; *FLE* = femtosecond laser energy; *IOP* = intraocular pressure; *ISV* = index of surface variance; *OBL* = opaque bubble layer; *RST* = residual stromal thickness

### Predictive performance and associated factors for OBL occurrence

Among the 15 classification algorithms employed for predicting OBL occurrence, the Extra Trees model demonstrated the most favorable predictive performance across the reported evaluation metrics. On the internal hold-out test set (44 eyes; 15 OBL-positive and 29 control eyes), the ET model yielded an ACC of 0.820 (95% CI: 0.797–0.842) and an AUC of 0.885 (95% CI: 0.871–0.899); the Kappa coefficient reached 0.658, indicating moderate-to-good agreement between the model predictions and observed outcomes. The performance of the five top-ranked classification algorithms is summarized in Table 2, and the receiver operating characteristic curve for the representative model is shown in Additional file 2.

**Table 2:** Performance of the classification models for predicting intraoperative OBL occurrence

Top 5 algorithms	Training-set 10-fold cross-validation	Internal hold-out test set	Top 5 algorithms	Training-set 10-fold cross-validation
	ACC (95% CI)	AUC (95% CI)		ACC (95% CI)
Extra Trees classifier	0.833 (0.807–0.858)	0.893 (0.880–0.905)	0.820 (0.797–0.842)	0.885 (0.871–0.899)
Ridge classifier	0.797 (0.772–0.822)	0.843 (0.830–0.857)	0.789 (0.764–0.815)	0.806 (0.796–0.817)
Linear discriminant analysis	0.792 (0.766–0.817)	0.876 (0.866–0.886)	0.777 (0.751–0.802)	0.812 (0.803–0.822)
Logistic regression	0.785 (0.760–0.815)	0.872 (0.858–0.887)	0.772 (0.747–0.797)	0.799 (0.786–0.812)
CatBoost classifier	0.785 (0.766–0.805)	0.820 (0.811–0.828)	0.780 (0.760–0.800)	0.802 (0.792–0.811)

The training-set cross-validation values represent mean performance across the 10 held-out folds within the training set. Internal hold-out values were calculated after model selection on 44 eyes; their 95% CIs were obtained from 2000 stratified bootstrap resamples of the fixed test-set predictions

*ACC* = accuracy; *AUC* = area under the receiver operating characteristic curve; *CI* = confidence interval; *OBL* = opaque bubble layer

**Fig. 3:**
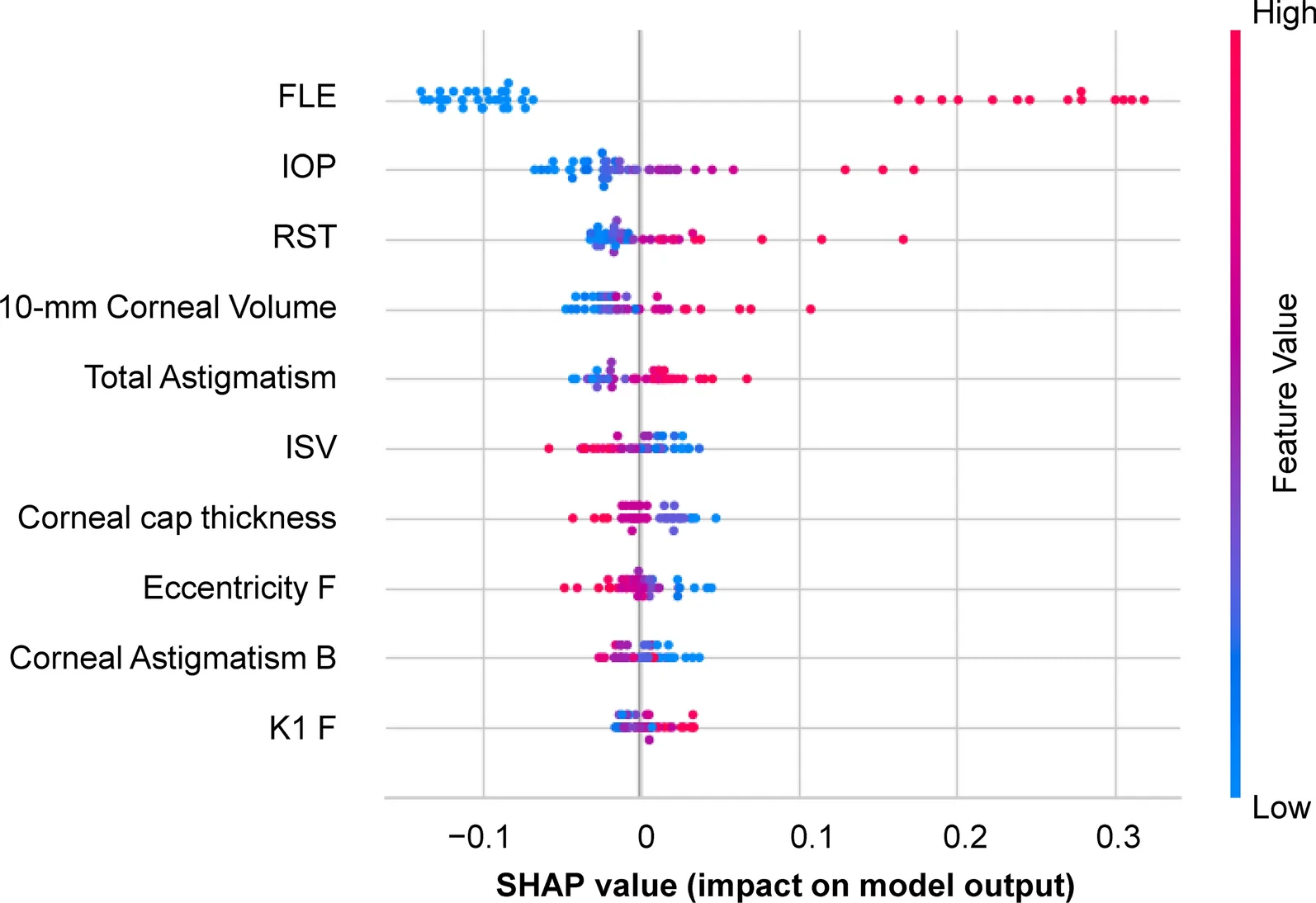
SHAP summary plots for the top 10 features in the best-performing OBL occurrence prediction algorithm. The higher the SHAP value for each feature, the higher the risk of OBL occurrence. B, posterior; F, anterior; FLE, femtosecond laser energy; IOP, intraocular pressure; ISV, index of surface variance; K1 flat keratometry; OBL, opaque bubble layer; RST, residual stromal thickness; SHAP, Shapley additive explanations

To further interpret the decision-making process of the Extra Trees model and quantify the contribution of individual preoperative parameters to OBL occurrence risk, we applied the SHAP approach for in-depth feature-importance analysis and association exploration. Figure 3 shows the SHAP summary plot for the prediction of OBL occurrence, which ranks predictive features based on their mean absolute SHAP values. The direction of the association between each feature and OBL occurrence risk was clearly distinguished by the SHAP summary plot: femtosecond laser energy, IOP, RST, 10-mm CV, total astigmatism, and anterior K1 showed positive correlations with OBL risk, where higher feature values corresponded to positive SHAP values and an elevated probability of intraoperative OBL formation. In contrast, ISV, corneal cap thickness, anterior surface numerical eccentricity and posterior corneal astigmatism exhibited negative correlations with OBL risk, with higher feature values leading to negative SHAP values and a reduced risk of OBL occurrence.

### Predictive performance and associated factors for OBL area

Among the 19 regression algorithms evaluated for predicting the relative OBL area (percentage of the quantified OBL area relative to the total posterior corneal surface area), the random forest regression (RFR) model achieved the most favorable fitting and predictive performance. On the internal hold-out test set of OBL-positive eyes (n = 15), the RFR model yielded an MAE of 2.89% (95% CI: 2.72% to 3.06%) and RMSE of 3.37% (95% CI: 3.25% to 3.48%). Table 3 summarizes the performance of the five top-ranked regression algorithms, and the residual diagnostic plot for the RFR model is shown in Additional file 3. The study cohort and dataset allocation within the two-stage modeling framework are summarized in Additional file 4.

**Table 3:** Performance of the regression models for relative OBL area during SMILE surgery

Top 5 algorithms	Training-set 10-fold cross-validation		Internal hold-out test set	
	MAE (95% CI)	RMSE (95% CI)	MAE (95% CI)	RMSE (95% CI)
Random forest regressor	0.0297 (0.028–0.0313)	0.0352 (0.0340–0.0364)	0.0289 (0.0272–0.0306)	0.0337 (0.0325–0.0348)
Extra trees regressor	0.0310 (0.0290–0.0330)	0.0363 (0.0353–0.0374)	0.0302 (0.0283–0.0321)	0.0343 (0.0333–0.0353)
Light gradient boosting machine	0.0321 (0.0301–0.0340)	0.0367 (0.0355–0.0379)	0.0311 (0.0292–0.0330)	0.0354 (0.0342–0.0365)
Orthogonal matching pursuit	0.0316 (0.0299–0.0333)	0.0376 (0.0361–0.0390)	0.0309 (0.0292–0.0327)	0.0367 (0.0352–0.0382)
Lasso least angle regression	0.0319 (0.0304–0.0333)	0.0398 (0.0384–0.0412)	0.0315 (0.0300–0.0331)	0.0388 (0.0374–0.0402)

The training-set cross-validation values represent mean performance across the 10 held-out folds within the training set. Internal hold-out values were calculated after model selection on 15 OBL-positive eyes; their 95% CIs were obtained from 2000 bootstrap resamples of the fixed observed-predicted pairs

*CI* = confidence interval; *MAE* = mean absolute error; *RMSE* = root mean square error; *SMILE* = small-incision lenticule extraction

**Fig. 4:**
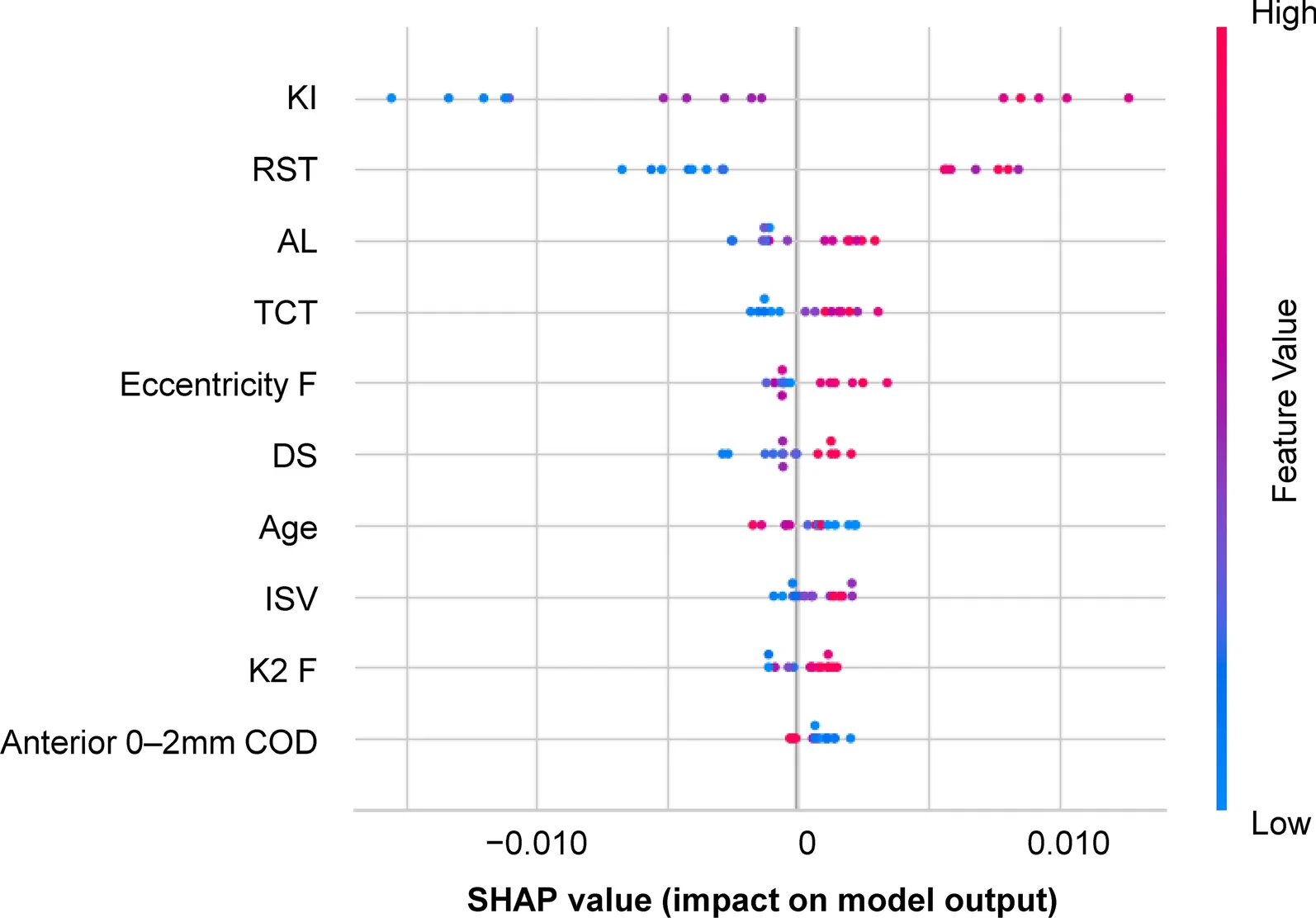
SHAP summary plots for the top 10 features in the best-performing relative OBL area prediction algorithm. The higher the SHAP value for each feature, the higher the relative area of the OBL. AL, axial length; COD, corneal optical density; DS, diopter sphere; F, anterior; K2, steep keratometry; KI, keratoconus index; OBL, opaque bubble layer; ISV, index of surface variance; RST, residual stromal thickness; SHAP, Shapley additive explanations; TCT, thinnest corneal thickness

SHAP analysis was further applied to quantify the contribution of each preoperative parameter to the relative OBL area and clarify the direction of their associations (Fig. 4). The direction of association indicated that age and COD (within the central 2-mm zone of the anterior 120-μm corneal layer) were negatively correlated with the relative OBL area, which means that higher values of these two parameters corresponded to a smaller predicted relative OBL area. In contrast, the remaining eight variables showed positive correlations with the relative OBL area, with higher feature values leading to a larger predicted relative OBL area. Notably, KI was the most influential variable.

## Discussion

Although OBL formation during SMILE is an important intraoperative complication, investigations into its associated factors remain relatively limited. Therefore, this study comprehensively evaluated the associations between preoperative parameters and OBL occurrence and severity, with the aim of developing an effective predictive model for intraoperative OBL risk. Some predictors, such as femtosecond laser energy and RST, are surgical-planning parameters rather than strictly preoperative biometric variables, suggesting that current models may be more suitable for risk assessment after an initial surgical plan has been generated.

Among the 10 key factors identified as being associated with OBL formation in this study, only a limited number have been previously reported. Both our findings and those of previous studies [10–12] indicated that a thicker RST and a thinner cap are associated with an increased likelihood of OBL formation. The underlying mechanism may be related to the denser arrangement of collagen fibers in the anterior corneal stroma [16, 17]. When femtosecond laser scanning is performed at a relatively shallow depth, the generated gas bubbles may be more likely to remain trapped within the compact anterior stroma, making dissipation challenging. Higher femtosecond laser energy was also identified as a model-associated factor; it may produce bubbles with larger volumes and longer lifespans, thereby intensifying bubble interactions and accumulation [18]. As these are model-derived associations rather than causal effects, the present findings do not justify changing femtosecond laser energy; a prospective evaluation is required before any parameter recommendations can be made.

Notably, this study identified CV in a diameter of 10 mm and IOP as important predictors associated with OBL formation. CV and central corneal thickness are generally considered parameters that fluctuate in parallel [19]. A larger CV in a diameter of 10 mm is often associated with greater corneal thickness that may be related to a relatively shallower scanning plane in which the collagen structure is denser, which could be associated with increased resistance to bubble dissipation [9]. A higher IOP is associated with greater corneal stiffness [20], which may impede the dissipation of gas bubbles. Given the close relationship between IOP and corneal biomechanics, further studies are warranted to investigate the role of corneal biomechanical parameters in OBL formation. Regarding corneal morphological parameters, we found that a lower ISV, smaller anterior surface eccentricity, smaller posterior corneal astigmatism, and smaller anterior K1 were associated with OBL occurrence. These findings suggest that corneas with regular surfaces and smaller overall curvatures, reflecting a flatter and spherical corneal shape, may have a higher likelihood of OBL formation.

Compared with previous studies that mainly reported associations between OBL and parameters such as corneal thickness, cap thickness, and laser-related factors, our findings were partly consistent in showing associations with RST, cap thickness, and femtosecond laser energy. However, the identification of 10-mm CV, IOP, corneal irregularity indices, and COD as model-identified predictors extends prior work by incorporating broader tomographic and densitometric information. The differences observed between studies may reflect variations in OBL definitions, surgical platforms and settings, case selection, sample size, and whether OBL was analyzed as a binary occurrence or as a quantitative area outcome. Unlike prior deep learning studies that mainly used intraoperative images to predict or generate OBL-related features, the present study focused on preoperative risk stratification by integrating routinely available ocular, tomographic, densitometric, and surgical-planning parameters in a two-stage ML framework for both OBL occurrence and relative OBL area.

Our clinical experience and previous study indicated that a larger OBL area is associated with increased difficulty in lenticule dissection and a higher incidence of intraoperative complications, such as lenticule tears [6]. Accordingly, we performed a quantitative analysis of the OBL area and comprehensively evaluated the associations between various preoperative parameters and OBL area. Interestingly, we found that higher KI, ISV, and anterior surface eccentricity values were associated with a larger OBL area. The elevation of these three parameters indicates increased overall corneal irregularity. We speculate that corneal irregularities may be related to dispersion of the femtosecond laser focus, allowing a broader region of the corneal tissue to absorb energy exceeding the threshold and potentially contributing to more extensive photodisruption. Consequently, the overall corneal irregularity reflected by a higher ISV and greater anterior surface eccentricity tended to be associated with an enlarged OBL area. This finding does not contradict the previously mentioned result that a lower ISV and smaller anterior surface eccentricity are associated with OBL formation. OBL formation is primarily determined by the retention of gas bubbles, whereas the OBL area is more dependent on the capacity for bubble diffusion and coalescence. Notably, this study is the first to incorporate COD into the analysis. We demonstrated that a lower COD within the central 2-mm zone of the anterior 120-μm corneal layer was associated with an increased OBL area. A lower COD indicates a relatively looser collagen fiber arrangement in the anterior stromal layer [21] with lower structural resistance, thereby providing more favorable conditions for bubble diffusion and coalescence.

This study has several limitations. First, this was a retrospective single-center study with a modest sample size, which may have introduced selection bias, and the 1:2 matched case-control design may have altered the natural prevalence of OBLs. Consequently, the predicted probabilities were not calibrated to the absolute clinical risk, and thus no probability threshold can be recommended. As age and sex defined the matching procedure and were excluded from the occurrence-classification predictors, the classification models could not learn interactions involving these variables. Matching also selectively restricted the control sample and truncated its natural covariate distribution, which may have reduced transportability to unmatched populations. Second, residual confounding from other clinical, anatomical, or surgical-planning factors cannot be excluded. Third, although 10-fold cross-validation and an internal hold-out test set were used, the relatively large number of candidate predictors, single random split, and absence of multicenter or temporally independent external validation may introduce overfitting and limit generalizability. The bootstrap CIs quantify sampling variability conditional on the fixed test-set predictions and do not propagate uncertainty from preprocessing, model fitting, tuning, or model selection; therefore, the point estimates and CIs may be sensitive to the particular split and should be interpreted descriptively. Fourth, although the two-stage framework restricted the area-regression task to OBL-positive eyes, the relative OBL area among affected eyes may remain skewed. Fifth, OBL classification and area quantification were based on intraoperative video frames and ImageJ thresholding, which may introduce measurement variability. Sixth, some intraoperative factors, such as docking quality, suction stability, ocular movement, and real-time procedural adjustments, were not quantitatively recorded and, therefore, not included in the models. Finally, several corneal structural and tomographic parameters may be correlated, which could affect the stability of SHAP-based feature importance. Thus, the SHAP results should be interpreted as model-derived associations rather than as causal mechanisms. Future consecutive multicenter studies with natural OBL prevalence, standardized OBL grading, repeated measurements, calibration assessments, external validation, and additional biomechanical parameters are needed to confirm and refine the study findings.

## Conclusions

In summary, the exploratory two-stage ML framework discriminated OBL occurrence and descriptive prediction of the relative OBL area within a single-center matched sample while identifying model-associated preoperative and surgical-planning factors. The predicted probabilities were not calibrated to absolute clinical risk, and thus no probability threshold or surgical-parameter recommendations can be made. The study findings are hypothesis-generating and require validation and recalibration in larger consecutive multicenter cohorts before approval for clinical use.

## Supplementary Information

Additional file 1

Additional file 2

Additional file 3

Additional file 4

## Data Availability

Data and analytical code are available upon reasonable request.

## Abbreviations

ACC: Accuracy
AL: Axial length
AUC: Area under the receiver operating characteristic curve
CI: Confidence interval
COD: Corneal optical density
CV: Corneal volume
IOP: Intraocular pressure
ISV: Index of surface variance
KI: Keratoconus index
K1: Flat keratometry
K2: Steep keratometry
MAE: Mean absolute error
ML: Machine learning
OBL: Opaque bubble layer
RFR: Random forest regression
RMSE: Root mean square error
RST: Residual stromal thickness
SD: Standard deviation
SE: Spherical equivalent
SHAP: Shapley additive explanations
SMILE: Small-incision lenticule extraction
