# Supplementary material for: Preoperative predictive factors for opaque bubble layer formation and area during small-incision lenticule extraction: predictive models based on machine learning
Source: Eye Vis (Lond). 2026 Sep 21;13:39. doi: 10.1186/s40662-026-00509-w (PMC13591720; doi:10.1186/s40662-026-00509-w)
Supplement: Supplementary file 1 [file 40662_2026_509_MOESM1_ESM.docx]

**Additional file 1.** Fifty-four ocular characteristics data between OBL group and its controls

| **Variables** | **Control** | **OBL** | ***P* value** |
| --- | --- | --- | --- |
| Relative OBL area (%) | 0.06 ± 0.05 | 7.66 ± 3.59 | < 0.001 |
| UCVA (logMAR) | 1.02 ± 0.28 | 1.04 ± 0.24 | 0.750 |
| IOP (mmHg) | 14.06 ± 2.23 | 15.99 ± 2.95 | < 0.001 |
| AL (mm) | 25.58 ± 0.88 | 25.49 ± 0.95 | 0.487 |
| DS (D) | −4.12 ± 1.47 | −4.41 ± 1.72 | 0.238 |
| DC (D) | −1.01 ± 0.77 | −0.68 ± 0.62 | < 0.001 |
| CDVA (logMAR) | 0.00 ± 0.01 | 0.00 ± 0.01 | 0.350 |
| Corneal cap diameter (mm) | 7.44 ± 0.13 | 7.45 ± 0.14 | 0.850 |
| Corneal cap thickness (μm) | 117.29 ± 6.71 | 117.36 ± 7.12 | 0.778 |
| RST (μm) | 321.58 ± 25.68 | 340.67 ± 34.94 | < 0.001 |
| FLE, index | 27.14 ± 0.35 | 27.60 ± 0.49 | < 0.001 |
| COD |  |  |  |
| Anterior layer |  |  |  |
| 0–2 mm | 17.14 ± 5.37 | 16.68 ± 4.95 | 0.829 |
| 2–6 mm | 15.50 ± 4.74 | 15.15 ± 4.40 | 0.835 |
| 6–10 mm | 15.88 ± 5.25 | 15.96 ± 5.83 | 0.871 |
| 10–12 mm | 31.23 ± 8.48 | 31.21 ±9.60 | 0.985 |
| Total | 18.38 ± 4.89 | 18.13 ± 4.94 | 0.770 |
| Middle layer |  |  |  |
| 0–2 mm | 8.74 ± 2.30 | 8.43 ± 2.06 | 0.698 |
| 2–6 mm | 8.07 ± 2.02 | 7.84 ± 1.82 | 0.848 |
| 6–10 mm | 9.18 ± 2.48 | 9.03 ± 2.65 | 0.601 |
| 10–12 mm | 14.37 ± 4.43 | 13.89 ± 3.99 | 0.589 |
| Total | 9.55 ± 2.44 | 9.27 ± 2.26 | 0.698 |
| Posterior layer |  |  |  |
| 0–2 mm | 10.59 ± 2.69 | 10.39 ± 2.62 | 0.465 |
| 2–6 mm | 9.62 ± 2.32 | 9.44 ± 2.26 | 0.438 |
| 6–10 mm | 10.21 ± 2.92 | 10.29 ± 3.36 | 0.789 |
| 10–12 mm | 18.79 ± 5.27 | 18.49 ± 5.49 | 0.503 |
| Total | 11.42 ± 2.78 | 11.26 ± 2.85 | 0.642 |
| Full layers |  |  |  |
| 0–2 mm | 12.16 ± 3.40 | 11.84 ± 3.14 | 0.713 |
| 2–6 mm | 11.07 ± 2.98 | 10.81 ± 2.77 | 0.700 |
| 6–10 mm | 11.76 ± 3.49 | 11.76 ± 3.88 | 0.738 |
| 10–12 mm | 21.47 ± 5.44 | 21.20 ± 5.82 | 0.486 |
| Total | 13.12 ± 3.29 | 12.88 ± 3.27 | 0.663 |
| K1 F (D) | 42.28 ± 1.19 | 42.61 ±1.50 | 0.086 |
| K2 F (D) | 43.63 ± 1.32 | 43.74 ± 1.50 | 0.602 |
| Kmean F (D) | 42.94 ± 1.19 | 43.15 ± 1.47 | 0.286 |
| Corneal astigmatism F (D) | 1.36 ± 0.74 | 1.13 ± 0.64 | 0.022 |
| Eccentricity F (mm) | 0.55 ± 0.11 | 0.52 ± 0.11 | 0.026 |
| K1 B (D) | −6.04 ± 0.19 | −6.14 ± 0.23 | 0.005 |
| K2 B (D) | −6.41 ± 0.23 | −6.49 ± 0.23 | 0.025 |
| Kmean B (D) | −6.21 ± 0.20 | −6.30 ± 0.22 | 0.010 |
| Corneal astigmatism B (D) | 0.38 ± 0.14 | 0.36 ± 0.14 | 0.295 |
| Eccentricity B (mm) | 0.54 ± 0.11 | 0.53 ± 0.12 | 0.747 |
| corneal vertex thickness (μm) | 547.58 ± 23.30 | 565.19 ± 29.87 | < 0.001 |
| Pupil center corneal thickness (μm) | 548.17 ± 23.43 | 565.51 ± 29.77 | < 0.001 |
| Thinnest corneal thickness (μm) | 544.79 ± 23.18 | 561.90 ± 29.72 | < 0.001 |
| CV within 3 mm (mm^3^) | 3.96 ± 0.17 | 4.09 ± 0.22 | < 0.001 |
| CV within 5 mm (mm^3^) | 11.58 ± 0.49 | 11.98 ± 0.62 | < 0.001 |
| CV within 7 mm (mm^3^) | 24.90 ± 1.06 | 25.78 ± 1.28 | < 0.001 |
| CV within 10 mm (mm^3^) | 61.00 ± 2.73 | 63.25 ± 3.03 | < 0.001 |
| ISV | 18.01 ± 5.02 | 16.15 ± 4.55 | 0.011 |
| IVA | 0.12 ± 0.05 | 0.11 ± 0.05 | 0.388 |
| KI | 1.03 ± 0.02 | 1.02 ± 0.02 | 0.302 |
| CKI | 1.01 ± 0.01 | 1.01 ± 0.01 | 0.897 |
| IHA | 5.85 ± 4.33 | 4.87 ± 3.46 | 0.205 |
| IHD | 0.01 ± 0.01 | 0.01 ± 0.01 | 0.889 |

AL = axial length; B = posterior; CDVA = corrected distance visual acuity; CKI = central keratoconus index; COD = corneal optical density; CV = corneal volume; DC = cylindrical refraction; DS = spherical refraction; F = anterior; FLE = femtosecond laser energy; IHA = index of height asymmetry; IHD = index of height decentration; IOP = intraocular pressure; ISV = index of surface variance; IVA = index of vertical asymmetry; K1 = flat keratometry; K2 = steep keratometry; KI = keratoconus index; OBL = opaque bubble layer; RST = residual stromal thickness; UCVA = uncorrected visual acuity
