# Supplementary material for: Preoperative predictive factors for opaque bubble layer formation and area during small-incision lenticule extraction: predictive models based on machine learning
Source: Eye Vis (Lond). 2026 Sep 21;13:39. doi: 10.1186/s40662-026-00509-w (PMC13591720; doi:10.1186/s40662-026-00509-w)
Supplement: Supplementary file 2 [file 40662_2026_509_MOESM2_ESM.docx]

**Additional file 2.** Receiver operating characteristic curve of the Extra Trees classification model. The receiver operating characteristic curve shows the performance of the final Extra Trees model for classifying opaque bubble layer (OBL) occurrence in the internal hold-out test set (44 eyes; 15 OBL-positive eyes and 29 controls). The model achieved an area under the receiver operating characteristic curve (AUC) of 0.884 and accuracy of 0.818. The dashed diagonal line represents chance-level discrimination.
