# Supplementary material for: Preoperative predictive factors for opaque bubble layer formation and area during small-incision lenticule extraction: predictive models based on machine learning
Source: Eye Vis (Lond). 2026 Sep 21;13:39. doi: 10.1186/s40662-026-00509-w (PMC13591720; doi:10.1186/s40662-026-00509-w)
Supplement: Supplementary file 3 [file 40662_2026_509_MOESM3_ESM.docx]

**Additional file 3.** Diagnostic plot of the Random Forest conditional OBL-area regression model. The diagnostic plot was generated using predictions from the OBL-positive internal hold-out test set (15 eyes). Residuals, calculated as the observed value minus the predicted value, plotted against the predicted relative OBL area. The dashed horizontal line represents a residual of zero. OBL, opaque bubble layer.
