# Supplementary material for: Preoperative predictive factors for opaque bubble layer formation and area during small-incision lenticule extraction: predictive models based on machine learning
Source: Eye Vis (Lond). 2026 Sep 21;13:39. doi: 10.1186/s40662-026-00509-w (PMC13591720; doi:10.1186/s40662-026-00509-w)
Supplement: Supplementary file 4 [file 40662_2026_509_MOESM4_ESM.docx]

**Additional file 4.** Study cohort and dataset allocation within the two-stage modeling framework. The final matched analytic cohort comprised 216 eyes derived from 216 patients, including 72 OBL-positive cases and 144 age- and gender-matched controls at a 1:2 ratio. For stage 1, all 216 eyes were included in the OBL-occurrence classification task, with 172 eyes allocated to model training and 10-fold cross-validation and 44 eyes reserved as an internal hold-out test set. For stage 2, the conditional OBL-area regression task was restricted to the 72 OBL-positive eyes, with 57 eyes allocated to training and 10-fold cross-validation and 15 eyes reserved for internal hold-out testing. Preprocessing, model comparison, and hyperparameter tuning were exclusively conducted within the corresponding training sets, whereas the internal hold-out sets were reserved for final performance evaluation. OBL, opaque bubble layer.
